# Successful endoscopic submucosal dissection for early gastric cancer involving the pyloric ring using a combination of water and gel immersion with the tunneling method

**DOI:** 10.1055/a-2106-5470

**Published:** 2023-07-13

**Authors:** Takahiro Muramatsu, Tomoaki Tashima, Tsubasa Ishikawa, Rie Terada, Tomonori Kawasaki, Takao Itoi, Shomei Ryozawa

**Affiliations:** 1 Department of Gastroenterology, Saitama Medical University International Medical Center, Saitama, Japan; 2 Department of Pathology, Saitama Medical University International Medical Center, Saitama, Japan; 3 Department of Gastroenterology and Hepatology, Tokyo Medical University Hospital, Tokyo, Japan

When performing endoscopic submucosal dissection (ESD) for gastric cancer that straddles the pyloric ring, it can be difficult to dissect the distal side of the lesion and visualize the submucosal layer. Although several techniques have been reported 1 2 3 , no consensus has been reached as to the best method. Recently, endoscopic treatment with water or gel immersion has become popular 4 5 . This has several advantages, including improving the visual field, providing buoyancy, and maintaining the lumen at a low pressure. Herein, we describe successful ESD for early gastric cancer involving the pyloric ring using a combination of water and gel immersion in low-pressure endoscopy with a tunneling method ( Video 1 ).

**Video 1** Successful endoscopic submucosal dissection of an early gastric cancer involving the pyloric ring using a tunneling technique and a combination of water and gel immersion.


The patient was an 84-year-old woman with early gastric cancer (25 mm in diameter) involving the pyloric ring ( Fig. 1 a–c ). The distal side of the lesion could not be observed in the antegrade position ( Fig. 1 d, e ), or with retroflexion at the duodenal bulb ( Fig. 1 f ). We removed the gas from the lumen and filled it with water, and the distal side of the lesion became visible under the low-pressure conditions ( Fig. 2 a, b ). First, a mucosal incision was made at the distal side to create an end point ( Fig. 2 c–e ). Unexpected bleeding occurred during the mucosal incision, and complete hemostasis was achieved under gel immersion. Second, a tunnel was created from the proximal side of the lesion toward the end point using a mixture of water and gel immersion ( Fig. 2 f, g ). The view of the tunnel was clear, which was also because of the presence of water and gel. Finally, submucosal dissection was performed to widen the tunnel, and this was followed by en bloc resection ( Fig. 2 h–j ).

**Fig. 1:**
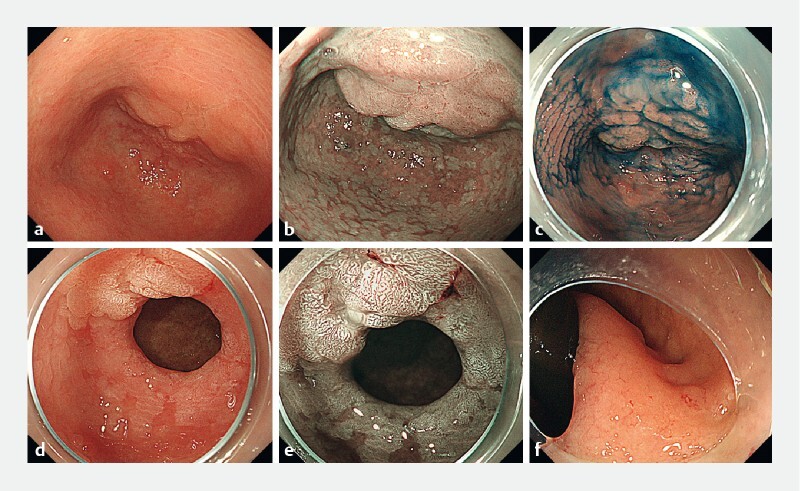
Endoscopic images showing: **a–c** a flat elevated lesion (0-IIa) of 25 mm in diameter involving the pyloric ring; **a** on white-light endoscopy; **b** on narrow-band imaging (NBI); **c** with indigo carmine dye; **d, e** that it was not possible to see the distal side of the lesion when using the transparent hood; **f** after retroflexion at the duodenum bulb, that the distal side of the lesion was not observable.

**Fig. 2:**
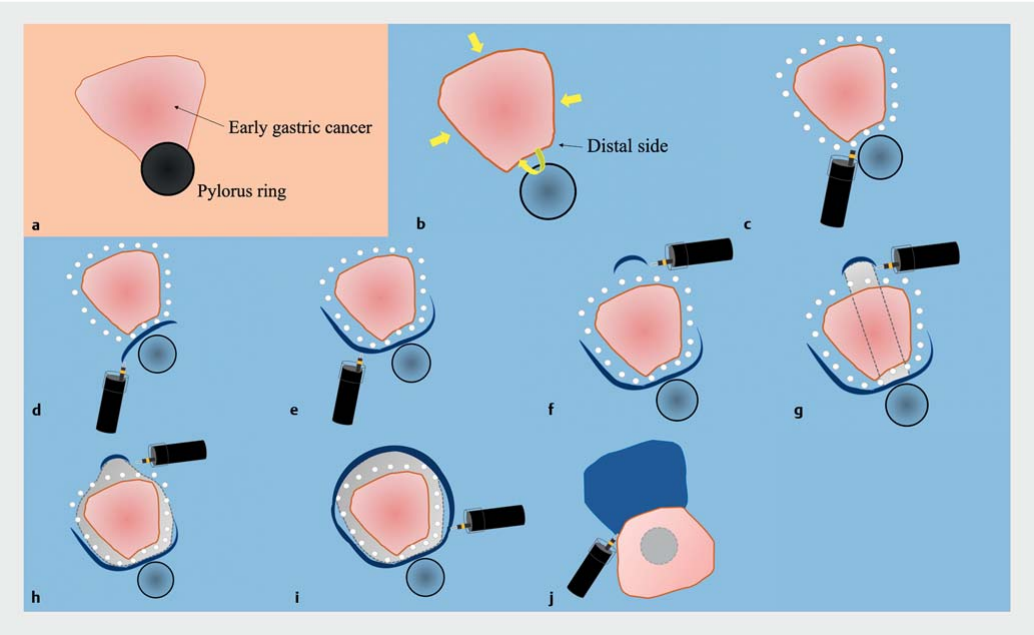
Schematic of the steps in resection of the lesion using the tunneling method with a combination of water and gel immersion in low-pressure endoscopy showing; **a** the appearance of the lesion with gas in the lumen; **b** visibility of the distal edge of the lesion on underwater view; **c** markings made around the lesion; **d** an initial mucosal incision made at the distal edge of the lesion; **e** widening of the mucosal incision; **f** creation of the tunnel entrance; **g** extension of the tunnel toward the end point; **h** submucosal dissection to widen the tunnel; **i** a full circumferential incision; **j** dissection of the remaining submucosal layer.

The tunneling method using low-pressure endoscopy with water and gel immersion overcomes the difficulties experienced in this situation. This method with its low pressure stabilizes the patientʼs condition, improves operability, and enables safe resection of lesions that are straddling the pyloric ring.

Endoscopy_UCTN_Code_TTT_1AO_2AG

## Notes

Endoscopy E-Videos

https://eref.thieme.de/e-videos
*E-Videos* is an open access online section of the journal *Endoscopy* , reporting on interesting cases and new techniques in gastroenterological endoscopy. All papers include a high-quality video and are published with a Creative Commons CC-BY license. Endoscopy E-Videos qualify for HINARI discounts and waivers and eligibility is automatically checked during the submission process. We grant 100% waivers to articles whose corresponding authors are based in Group A countries and 50% waivers to those who are based in Group B countries as classified by Research4Life (see: https://www.research4life.org/access/eligibility/ ). This section has its own submission website at https://mc.manuscriptcentral.com/e-videos
